# Draft Genome Sequence of *Bacillus ginsengihumi* Strain M2.11 with Phytase Activity

**DOI:** 10.1128/genomeA.00851-15

**Published:** 2015-08-13

**Authors:** Anna A. Toymentseva, Aliya D. Suleimanova, Eugenia A. Boulygina, Sergey V. Kazakov, Daria S. Baranova, Alina I. Akhmetova, Ayslu M. Mardanova, Margarita R. Sharipova

**Affiliations:** a Interdisciplinary Center for Proteomics Research, Kazan (Volga region) Federal University, Kazan, Russia; b Laboratory of Biosynthesis and Bioengineering of the Enzymes, Institute of Fundamental Medicine and Biology, Kazan (Volga region) Federal University, Kazan, Russia; c Computer Technologies Laboratory, Information Technologies, Mechanics & Optics University, St. Petersburg, Russia

## Abstract

This paper announces the genome sequence of *Bacillus ginsengihumi* strain M2.11, which has been characterized as a strain which produces the enzyme with the ability to degrade phytase. The genome of the strain M2.11 is 3.7 Mb and harbors 3,082 coding sequences.

## GENOME ANNOUNCEMENT

Phytases (myo-inositol hexakisphosphate phosphohydrolase) are valuable enzymes that recently found extensive use as feed additives. Phytases hydrolyze phytate into the less phosphorylated myo-inositol derivatives and inorganic phosphate (1). Thereby, such enzymes can improve mineral absorption, enhancing amino acid availability in feed for monogastric animals (2–4). Most of the microbial phytases are intracellular enzymes (5), but fungi (6) and bacteria of the genera *Enterobacter* (7) and *Bacillus* produce secreted phytases (8). Bacillary phytases are thermostable (>50% of its initial activity remained after incubation at 80°C for 10 min in the presence of Ca^2+^ ions) (9) and resistant to proteolytic degradation (10), which makes these enzymes prospective for application in industry. In this paper, we report on the sequencing of the genome of *Bacillus ginsengihumi* strain M2.11. This bacterium was isolated on phytase-screening medium from a forest soil sample in the Republic of Tatarstan, and its phytate-degrading enzyme was isolated and characterized (11).

16S rRNA gene sequencing was used for the identification of the M2.11 isolate. Thereby, strain M2.11 was classified as *B. ginsengihumi*. The draft genome sequence of strain M2.11 was obtained by using the 454-GS Junior platform (Roche), which was performed by the Interdisciplinary Center for Collective Use of Kazan Federal University, Russia. We obtained 99,708,428 total base pairs, with an average coverage of 25×. Through the *de novo* genome assembly using the SPAdes 3.1.0 software (12), we obtained 140 contigs with an *N*_50_ of 58,040 bp and the largest contig length of 200,339 bp. The estimated genome size was ~3.7 Mbp, with an average G+C content of 36.16%.

Genome annotation by the PGAAP pipeline (13) resulted in the identification of 3,082 coding sequences, 34 rRNA operons, and 88 tRNAs. The Rapid Annotations using Subsystems Technology (RAST) server (14) was also used for subsystem descriptions. The annotation identified genes involved in phosphorus assimilation: *pho* operon, polyphosphate kinase, exopolyphosphatase, pyrophosphate-specific outer membrane porin, phosphate-specific outer membrane porin, phosphate ABC transporter, and genes of alkaline phosphatase synthesis. Genes associated with phytase activity were not found, even though M2.11 has the ability to produce this enzyme. RAST analysis also showed the presence of several coding sequences (CDSs) with predicted antibiotic resistance functions, which was phenotypically confirmed by antibiotic sensitivity tests. In addition, the RAST server identified the following genes: 5 genes of osmotic stress, resistance genes, 37 genes of oxidative stress, and 19/3 genes of heat/cold shock. The complete sequencing of *B. ginsengihumi* M2.11 allows the prediction of physiological characteristics of the strain and the ability to use these data to create favorable conditions for strain cultivation.

### Nucleotide sequence accession numbers.

This whole-genome shotgun project has been deposited at GenBank under the accession no. JRUN00000000. The version described in this paper is version JRUN00000000.1.
